# Imaging presentation of pancreatic neuroendocrine neoplasms

**DOI:** 10.1007/s13244-018-0658-6

**Published:** 2018-10-09

**Authors:** Valentina Ciaravino, Riccardo De Robertis, Paolo Tinazzi Martini, Nicolò Cardobi, Sara Cingarlini, Antonio Amodio, Luca Landoni, Paola Capelli, Mirko D’Onofrio

**Affiliations:** 1 Department of Radiology, University Hospital G.B. Rossi, University of Verona, Verona, Italy; 2 Department of Radiology, Hospital Morgagni Pierantoni, Via Carlo Forlanini 4, 47121 Forlì, FC Italy; 3 Department of Radiology, Hospital “Casa di Cura Dott. Pederzoli”, Peschiera del Garda, Verona, Italy; 4 Department of Oncology, University Hospital G.B. Rossi, University of Verona, Verona, Italy; 5 Department of Gastroenterology, University Hospital G.B. Rossi, University of Verona, Verona, Italy; 6 Department of Surgery, University Hospital G.B. Rossi, University of Verona, Verona, Italy; 7 Department of Pathology, University Hospital G.B. Rossi, University of Verona, Verona, Italy

**Keywords:** Pancreatic neuroendocrine tumors, Pancreatic neuroendocrine neoplasms, P-NEN

## Abstract

**Abstract:**

Pancreatic neuroendocrine neoplasms (P-NENs) are the second most common solid pancreatic neoplasms. P-NENs have a wide range of imaging features presentations and they can be detected with typical and atypical imaging presentations. Typical and atypical appearances can be explained by pathologic correlations. P-NENs are generally hypervascular lesions, showing a typical enhancement behavior after contrast media injection during imaging methods, but they could also have different imaging features, creating some difficulty in differential diagnosis. For this reason, radiologists should be aware of different imaging presentations of these neoplasms. Radiological evaluation has a critical role in P-NENs identification, characterization, and staging of these neoplasms, especially in those cases in which surgery is the treatment of choice. The present paper shows, indicating the underlying pathologic correlations, typical and atypical presentations of NENs.

**Key Points:**

• *P-NENs have a wide range of imaging features presentations, typical and atypical.*

• *Pathology could help in better understanding the typical P-NENs appearance at imaging.*

• *P-NENs are generally hypervascular lesions.*

• *Radiological evaluation has a critical role in P-NENs identification and management.*

• *Radiologists should know every type of different imaging presentation of P-NENs to better diagnose these kinds of lesions.*

## Introduction

Pancreatic neuroendocrine neoplasms (P-NENs) have a wide range of imaging features presentations. They can show different typical and atypical, common and uncommon, rare and very rare imaging presentations. Typical appearances can be explained by pathologic correlations and multimodalities approaches (US, CT, MRI, EUS, and PET) can be used to better show these features.

A radiologist has to know every possible imaging presentations range to improve the diagnosis and, therefore, management and treatment of P-NENs.

P-NENs are the second most common solid pancreatic neoplasms, after the first pancreatic ductal adenocarcinoma. Nevertheless, they are rare neoplasms, accounting for 1–2% of all pancreatic lesions [1]. Even though they are rare tumors, in the last 20–30 years, their incidence has significantly increased more than twice, due to diagnostic imaging improvements and to medical knowledge increase [2–4]. Most of the time, they are sporadic tumors and they are solitary, whereas sometimes they are part of hereditary syndromes, such as multiple endocrine neoplasia type 1 (MEN1), Von Hippel–Lindau (VHL), neurofibromatosis type 1 (NF1), and tuberous sclerosis complex (TSC), and, in these hereditary syndromes, they present more frequently as multifocal lesions [1].

P-NENs can be divided into two categories, based on patients’ symptoms complained: functioning, if they produce and release hormones with different syndromes, depending on the produced molecule type, and non-functioning, in case of tumors inactivity. Non-functioning lesions are more frequent than functioning ones, accounting for two-thirds of all P-NENs [1, 2, 5–7]. However, in recent years, the small non-functioning lesions have been diagnosed incidentally with increased frequency in asymptomatic patients, due to imaging techniques improvements.

## Pathology

**Fig. 1:**
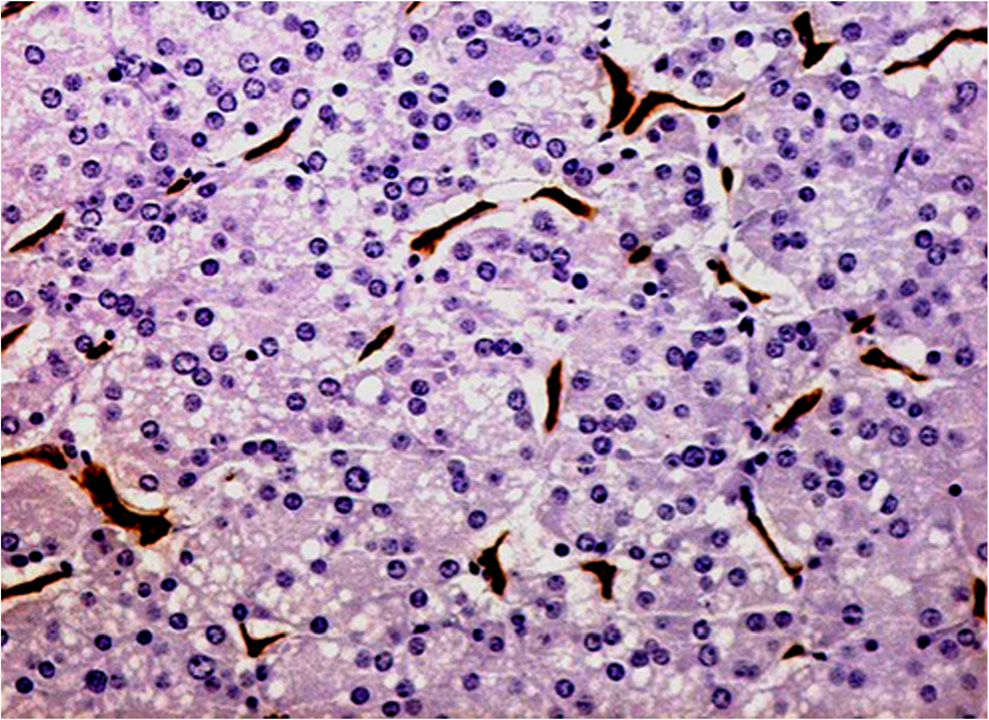
Pancreatic neuroendocrine neoplasm (P-NEN). Histological analysis: neuroendocrine neoplasm (NEN) showing high cellularity during hematoxylin and eosin staining and high intralesional vascular network demonstrated by CD34 immunohistochemical staining

**Fig. 2:**
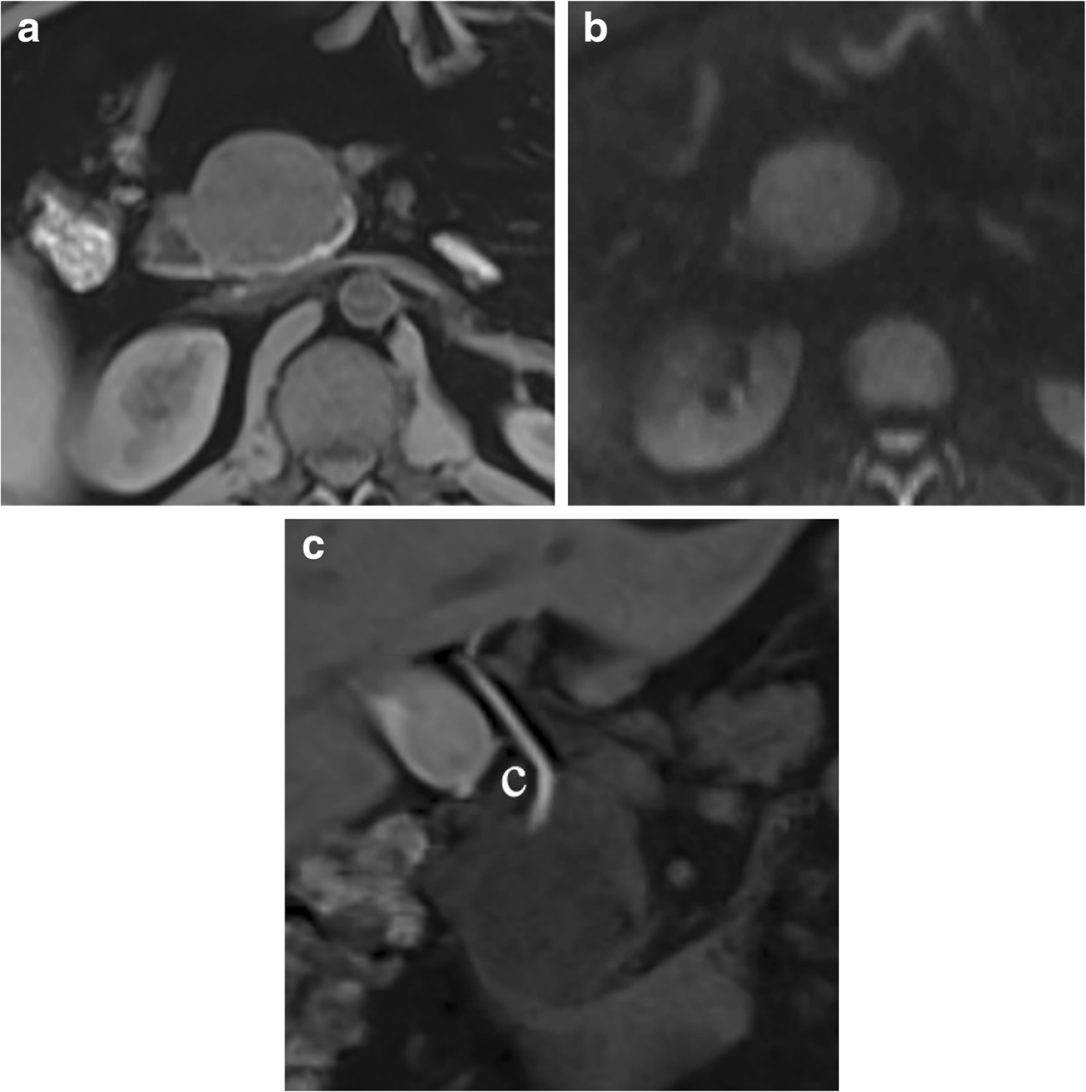
Capsulated NEN. MRI study: the pancreatic head lesion is slightly hypointense on T1-weighted fat-saturated axial images (**a**) and presents diffusion restriction (**b**) on DWI (*b* = 800). In the late hepatospecific phase (**c**) with contrast medium (Gd-BOPTA), the common bile duct (*C*) is clearly visible and not dilated, since it is displaced but not compressed by the pancreatic head mass

The typical P-NEN is rich in small vessels with high cellularity and poor fibrotic stroma (Fig. 1), usually giving a homogenous macroscopic appearance, with, in most cases, a greater consistency than the adjacent pancreatic parenchyma. Calcifications and necrosis can be present, especially in large masses [1, 2]. A rich vascularization is typical of the large majority of P-NENs, as previously stated, and it is responsible for the hypervascular typical aspect in imaging studies with contrast media. The majority of P-NENs present as a solitary, solid, delineated mass with a rounded or multilobulated margins and a sharp delimitation from the surrounding parenchyma; sometimes, there could be the presence of a fibrotic pseudo-capsule that partially or entirely surrounds the tumor; they are rarely encapsulated. P-NENs very often show expansive growth pattern with compression of adjacent structure, such as the main pancreatic and/or biliary ducts (Fig. 2). However, depending on the aggressiveness, P-NENs can grossly show features of malignancy with evident invasive growth pattern infiltrating adjacent ducts, structures, and organs [1, 8].

The tumor grade is one of the most important prognostic factors of neuroendocrine tumors, underlying the importance of knowing this data, by means of histological analysis. With increase of the tumor grade, the prognosis is lower.

In the 2017, the NENs WHO classification was revised and NENs have been classified according to the ENETS grading system, which is based on the proliferative activity of the neoplasm:

- NEN G1: Ki67 < 3% and/or < 2/10 mitosis 10/HPF; well differentiated
- NEN G2: Ki67 > 3% and < 20% and/or 2–20 mitosis 10/HPF; well differentiated
- NEN G3: Ki67 > 20% and/or > 20 mitosis 10/HPF; well differentiated
- NEC (neuroendocrine carcinoma) G3 small cells: Ki67 > 20% and/or > 20 mitosis 10/HPF; poorly differentiated
- NEC G3 large cells: Ki67 > 20% and/or > 20 mitosis 10/HPF; poorly differentiated

## P-NENs classification

**Fig. 3:**
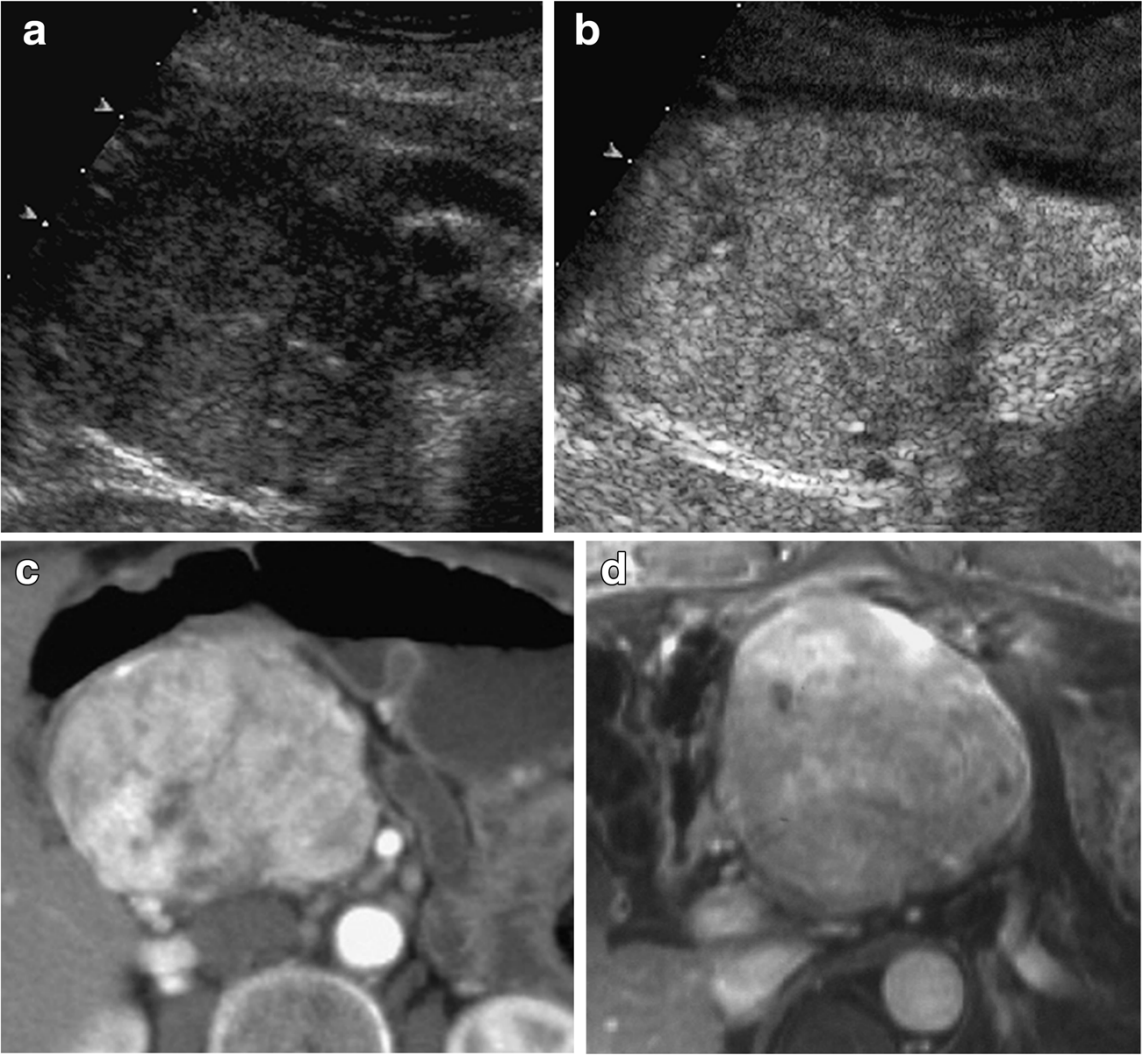
Non-functioning NEN. US and CEUS examinations: large hypoechoic mass (**a**) with small calcifications in the pancreatic head, causing upstream dilation of the Wirsung duct. This lesion is inhomogeneously hypervascularized at CEUS (**b**). CT examination: the pancreatic mass appears inhomogeneously hyperenhancing (**c**) in respect to the surrounding pancreatic parenchyma on dynamic phases. Dynamic MRI: inhomogeneous hypervascularity (**d**) of the pancreatic head mass

The most common characteristic of P-NENs is that, generally, they are hypervascular, showing a typical enhancement behavior after contrast media injection during several imaging methods (Fig. 3).

As previously stated, these neoplasms are mainly subdivided into two large groups (functioning and non-functioning), with different main features.

Functioning P-NENs are usually diagnosed in younger patients compared to the non-functioning P-NENs (55 years vs. 59 years), and the first type are usually smaller in dimension (< 3 cm) and usually non-metastatic at the time of diagnosis. However, the non-functioning neoplasms are generally the majority of P-NENs, accounting for about 40–90% of cases [2, 9]. Usually, functioning P-NENs present early with clinical manifestations related to the produced hormones, so, often, patients with P-NENs undergo imaging studies with a strong suspicion of disease.

**Fig. 4:**
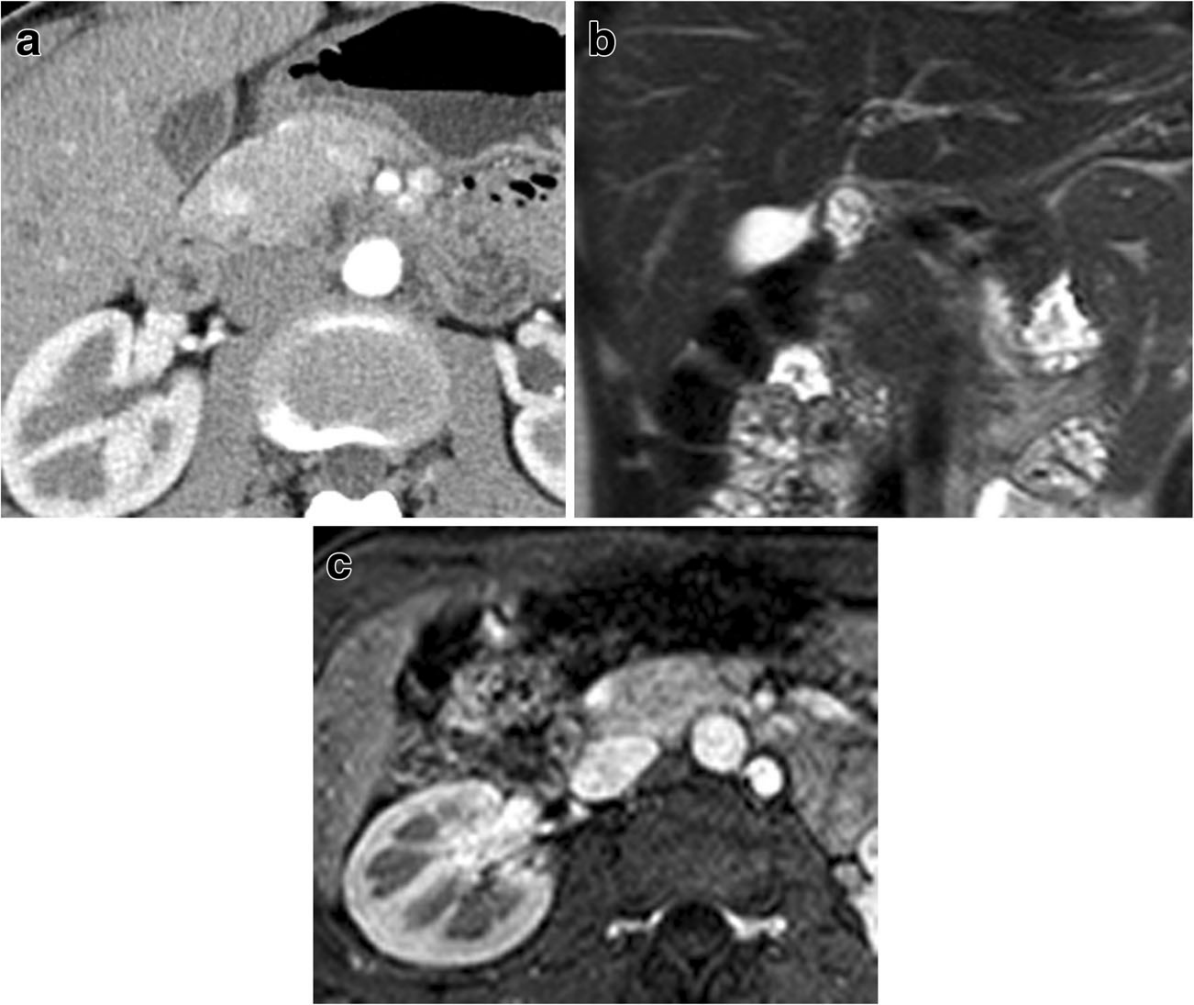
Insulinoma. CT: a small insulinoma appearing as a hypervascular hyperdense nodule (**a**) in the pancreatic head. MRI: the small insulinoma is well detectable as a hyperintense nodule (**b**) on T2-weighted coronal images and hypervascular (**c**) in the dynamic phase

Insulinomas are the most common functioning P-NENs and they account for about 60% of these neoplasms. Insulinomas are slightly prevalent in middle-aged (V–VI decades) women, and they are usually ubiquitarian within the gland, maybe with a slight prevalence in the body-tail. Usually, they have a benign course and behavior (85–99%) and they are often small (Fig. 4), smaller (90% < 2 cm) than other types. However, they can also be bigger and they could have a malignant behavior in some cases. At the time of clinical presentation, about half of cases are smaller than 1.5 cm [1, 7]. Insulinomas are usually solitary, but they could be multifocal in the majority of patients with MEN1 disease.

The second most common type of functioning P-NENs are gastrinomas, accounting for about 20% of cases. Gastrinomas are slightly prevalent in middle-aged (V decade) men and they are usually located in the “gastrinoma triangle”, defined as the anatomical area among the junction of the cystic duct and common bile duct, the second and third duodenal portions, and the pancreatic head and neck. They tend to have a malignant course, accounting for about 60–65% of cases, and they are often multiple (about 60% cases, of which 20–60% in MEN1 disease) [1, 10].

Other histological types are somatostinomas, glucagonomas, ACTH-omas, and VIP-omas, accounting for a small percentage of P-NENs, about 20%, and their behavior can be atypical as a rule, so they tend to be bigger, malignant, and metastatic at diagnosis most of the time [1, 7, 10–12].

**Fig. 5:**
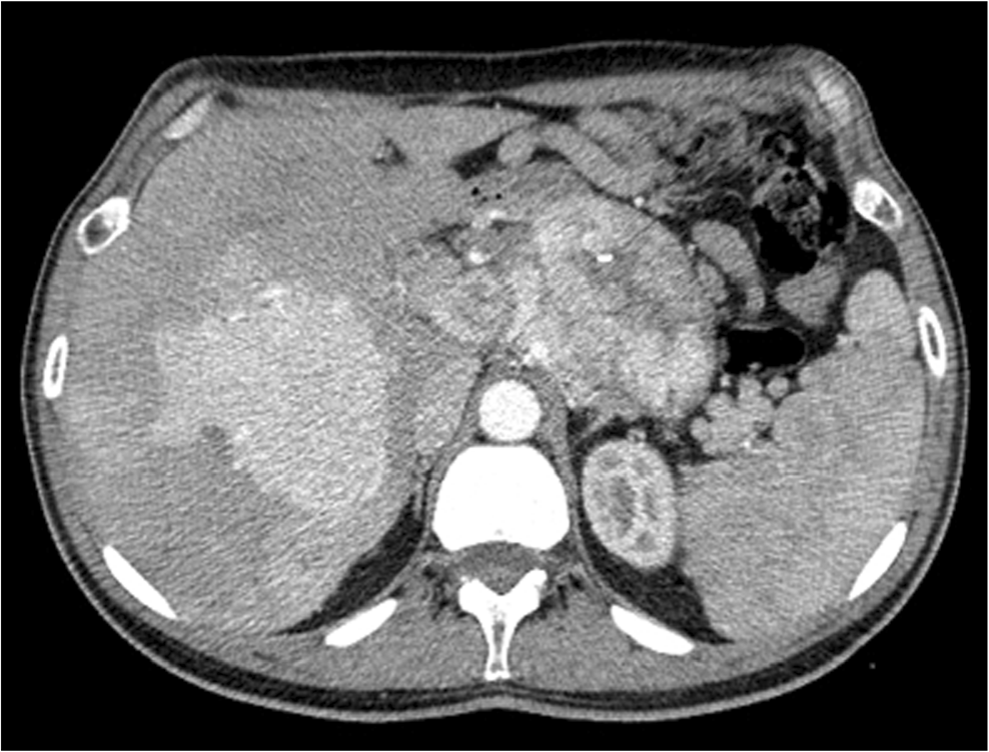
Non-functioning NEN. CT examination: huge inhomogeneously hypervascular pancreatic body-tail mass with necrotic areas and intralesional calcifications. Liver metastatic involvement is present

Non-functioning NENs account for the majority of P-NENs. They tend to be larger (Fig. 5) than functioning ones, ranging from 1 to 20 cm in diameter, and they usually show a higher malignancy rate, up to 90%, with infiltration of retroperitoneal structures, causing pain and other symptoms due to mass effect at the time of presentation. As a consequence, they are more often diagnosed at an advanced stage than functioning tumors. Non-functioning lesions frequently have an inhomogeneous aspect mainly due to necrotic areas and calcifications. They are predominantly characterized by an expansive growth pattern; therefore, they are usually clinically silent until adjacent viscera and structures are involved [1, 7].

As previously stated, small non-functioning P-NENs are being incidentally discovered with increasing frequency in asymptomatic patients, due to recent advances in imaging methods and an increasing number of imaging abdominal scans and studies [1, 13, 14].

## Typical imaging presentations

During imaging, P-NENs usually present as a solid hypervascular mass (Fig. 3).

During US, P-NENs are usually well-marginated hypoechoic masses with sharp margins, more or less homogeneous due to their size, which can vary. Doppler study could show small vessels within lesions. These neoplasms typically appear hypervascular (Fig. 3) during contrast-enhanced ultrasound (CEUS), and they usually show a rapid and intense enhancement in the early contrast-enhanced phase [1, 15, 16]. In bigger lesions, such as non-functioning types, necrotic intralesional areas can be seen within the rapid intense enhancement in the early dynamic phase at CEUS. Non-functioning P-NENs usually have a larger size than functioning P-NENs, which also justifies their tendency to contain necrosis and hemorrhage, giving them a typical presentation at imaging before and after contrast injection. Small calcifications, central necrotic or hemorrhagic, or cystic degeneration can be better identified during ultrasound harmonic imaging within P-NENs [1, 6, 17].

During CT, P-NENs typically present as more or less homogeneously isodense or slightly hypodense, well-defined masses during the pre-contrast phase, but they can also be inhomogeneous if larger. After contrast injection, during the arterial phase, they tend to be homogeneous or inhomogeneous hyperdense (Fig. 3), generally well depictable with respect to the adjacent parenchyma, and hyperdense or isodense during the portal phase, due to their vascularization. Very small intralesional calcifications can be presented in particular in the largest non-functioning lesions, which can also be inhomogeneous (Fig. 5), due to the presence of hypodense necrotic areas [1, 14, 18–20].

At MRI, they usually present as well-defined rounded lesions, homogeneously hypointense on T1-weighted sequence and hyperintense on T2-weighted sequence, better visualized with fat-suppression techniques. In case of bigger dimensions, they could be more inhomogeneous.

Diffusion-weighted imaging (DWI) may be useful in the study of P-NENs because, usually, they have clear restriction signal during DWI (Fig. 2) due to the high cellularity. DWI may help in the detection of P-NENS, especially for localizing non-hypervascular neoplasms [21]. It has been reported that P-NENs with different grades of differentiation can be distinguished evaluating ADC maps: poor-differentiated P-NENs have significantly lower mean ADC values compared to normal pancreatic tissue and to well-differentiated pancreatic neuroendocrine tumors [22–24]. DWI MR could predict pancreatic neuroendocrine tumor grade, as reported in the literature, playing a role in tumor prognostic stratification at the beginning of the clinical history of the patient [25]. After intravenous contrast administration, they typically appear hypervascular (Fig. 3) during the arterial phase and isointense or slightly hyperintense during the portal phase, more or less homogeneous related with their dimensions [1, 18, 19, 26].

Imaging methods can have a role in the detection, characterization, and staging of P-NENs. The staging of these tumors is important to choose the better treatment plan.

Metastases from P-NENs tend to resemble imaging features of primary tumors, usually showing enhancement after contrast media injection.

## Atypical imaging presentations and variants

P-NENs could show atypical behavior. The most common atypical presentations are the hypoenhancing pattern, the intravessels growth, the intraductal growth, and the cystic and calcified variants [22].

**Fig. 6:**
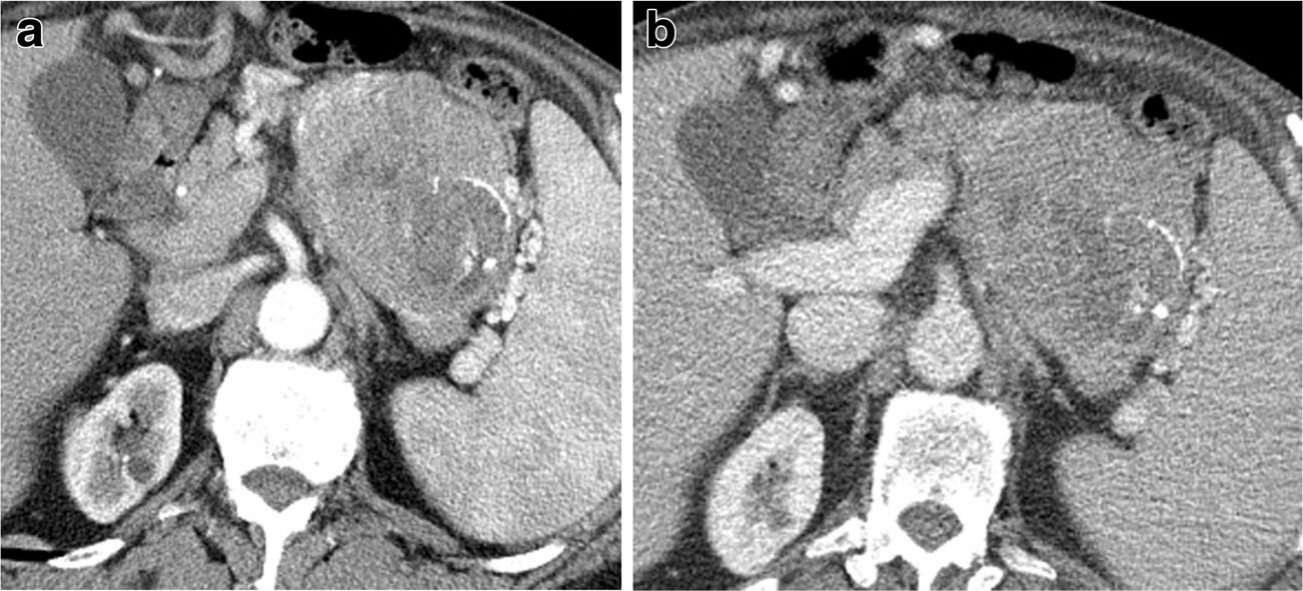
Non-functioning NEN. CT examination: huge inhomogeneously hypovascular pancreatic body-tail mass with necrotic areas and intralesional calcifications studied in the pancreatic (**a**) and venous (**b**) phases

P-NENs sometimes appear hypovascular (Fig. 6) after contrast media injection and/or with progressive enhancement in the portal and late phases, causing some problems in differential diagnosis, mimicking ductal adenocarcinoma [1]. Actually, hypovascular P-NENs are not so rare, reaching 49% in a group of patients with NENs in the clinical records of Jeon et al. [27]. This appearance is directly related to the amount of dense and hyalinized stroma within the tumor, and to the small lesion dimension or of its vascular network [22, 28, 29]. It is crucial to carefully evaluate all the other imaging characteristics (size, margins, growing pattern, and main pancreatic duct aspect) in order to carry out an accurate differential diagnosis and to differentiate them from ductal adenocarcinoma, because these two entities may have a completely different management and, last but not least, a completely different prognosis [22].

In this variant, the involvement of the main pancreatic duct is more frequent, with also the presence of the “double-duct sign” if there is a concomitant involvement of the common bile duct due to a lesion occurring in the pancreatic head. This tumor variant at imaging is very close to the expected picture of a ductal adenocarcinoma, especially if it is small in dimension, implicating the cytological or histological confirmation mandatory in every case [1]. At imaging, these fibrous P-NENs have atypical features, appearing hypovascular in the arterial phase at CT and MRI and with a possible progressive enhancement in the other phase due to the presence of fibrosis within lesions, except during CEUS due to the microbubble contrast media inner characteristics. Moreover, during MRI, it could lack the typical high hyperintensity on T2-weighted images, appearing isointense or slightly hyperintense on T2-weighted sequences, and, also, the strong restriction on DWI could be missed, causing mistakes in the differential diagnosis.

**Fig. 7:**
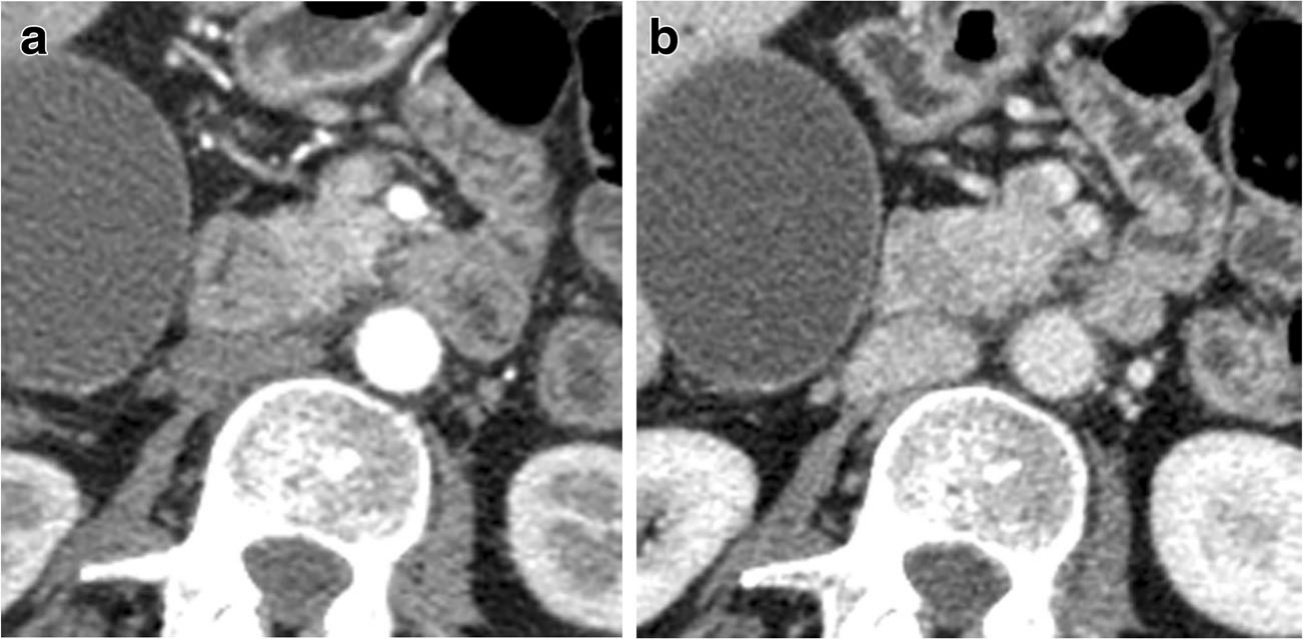
Non-functioning NEN. CT examination: small hypervascular pancreatic head mass irregular in shape invading the superior mesenteric vein with a small neoplastic thrombus studied in the pancreatic (**a**) and venous (**b**) phases

The intraductal growing and the intravessels growing are characterized by a colonization of their lumen with an endoluminal neoplastic cells growth. This growth type is a typical characteristic feature of P-NENs (Fig. 7) and they are very rare.

Intraductal location and growth of P-NENs may occur in two different scenarios. First, as part of a parenchymal pancreatic lesion strictly next to the main pancreatic duct that extends into the pancreatic duct and then grows along it, so the intraductal growth is connected to an extraductal lesion [1, 30, 31]. Second, the rarer setting of a NEN that originates within the main duct, fills the lumen as a polypoid mass, and grows along the duct. This type of lesion does not have a parenchymal component [32]. Usually, this behavior is shown by non-functioning neoplasms that could grow within the main pancreatic duct and may completely obstruct the lumen, causing an obstructive pancreatitis.

The intraductal growth could be very insidious at imaging because it could be small in dimension and could be masked by pancreatitis signs. In case of an intraductal lesion associated with intrapancreatic lesion, at imaging, it could be possible to see the intrapancreatic one. Whereas in the case of a lesion that is exclusively intraductal, this is very difficult to observe at imaging; in the majority of these cases, diagnosis is possible only after surgery. At US, lesions may appear hyperechoic and difficult to separate from adjacent pancreatic parenchyma. At CT, often, these lesions are not visible. MR could be useful due to cholangiopancreatography (MRCP) because it could be possible to see a filling defect because lesions could appear hypointense compared to surrounding fluid and it could be possible to see them enhance following the administration of contrast agent.

In case of vessels involvement, this particular growing pattern is usually confined to peri-pancreatic vessels, such as splenic and portal veins, producing a neoplastic thrombus, characterized by enhancement during contrast media imaging studies, quite the opposite of blood clot thrombus [33].

P-NENs may also present as a lesion growing around the main pancreatic duct, causing abrupt-type stricture with upstream dilatation and a normal-caliber duct downstream of the stricture [1, 34, 35]. These neoplasms are usually positive using serotonin and very small in dimensions, exhibiting highly fibrotic stroma, and, as a consequence, poor vascularization.

In 5–10% of cases, P-NENs could present a cystic degeneration, usually in non-functioning lesions, causing some difficulties in differential diagnosis. Usually, cystic NENs are larger, more often symptomatic, and more likely to be non-functional than solid NENs [36].

**Fig. 8:**
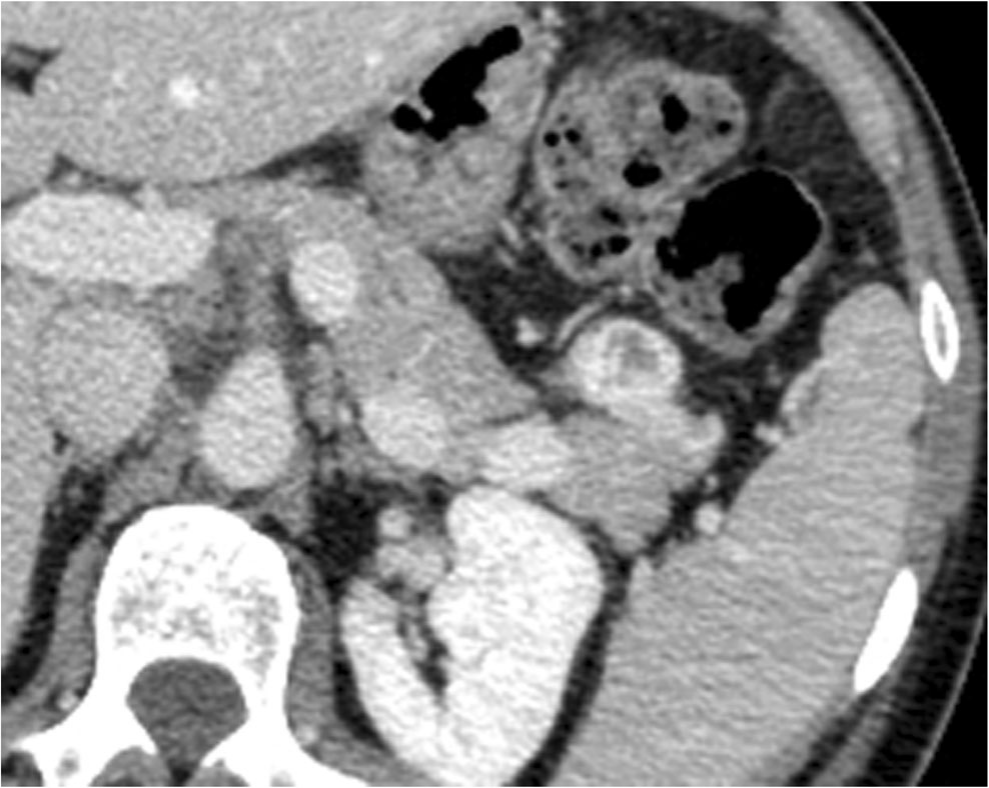
Cystic NEN. CT examination: small cystic exophytic pancreatic tail mass with hypodense central area surrounded by thick hypervascular rim

They present as a well-circumscribed cystic lesion, both unilocular and multilocular, usually with inner septa, with smooth margins and peripheral enhancement, usually on both arterial and portal phases, according to the hypervascular nature of P-NENs [37, 38]. The cyst is usually unilocular, in the center of the lesion, sometimes with septa, without enhancement post-contrast media injection, surrounded by a rim of neoplastic tissue hypervascular at imaging (Fig. 8).

During US, neuroendocrine cystic neoplasms are anechoic lesions delimited by a wall of variable thickness. In case of contrast media injection, this wall and eventual inner septa show enhancement.

During CT, usually, the cystic portion is hypodense, with wall and septa enhancement after contrast media injection.

CT is the most common initial imaging study in the evaluation of patients with cystic pancreatic lesions, whereas the gold standard in studying pancreatic cystic lesions is MR, due to its superior fluid and soft-tissue contrast, and it affords the best non-invasive means for the morphologic evaluation of cystic lesions of the pancreas. The MR signal is typically low in T1-weighted sequences, and high in T2-weighted sequences, with enhancement of wall and septa in post-contrastographic phases.

Calcifications may occur in P-NENs, unlike intra-tumor calcifications, which are not often seen in ductal adenocarcinoma. More commonly, calcifications occur within non-functioning and larger neoplasms [22, 38]. However, insulinoma may contain calcifications in up to 20% of cases. Overall, calcifications can be found in NENs in up to 16% of cases; these calcifications tend to be focal, coarse, irregular, and centrally located [22, 39].

A summary of the typical and atypical presentations of P-NENs is included in Table 1.

**Table 1:** Summary of typical and atypical aspects during imaging of pancreatic neuroendocrine neoplasms (P-NENs)

		US	CEUS	CT	MR
Typical P-NENs		Well-marginated hypoechoic with sharp margins	Hypervascular with rapid and intense enhancement	Isodense or slightly hypodense, well-defined, hyperdense in arterial phase, hyperdense or isodense in portal phase	Well-defined, hypointense on T1W and hyperintense on T2W (better conspicuity in FAT-SAT) Clear restriction signal at DWI Hypervascular in arterial phase and isointense or slightly hyperintense in portal phase
Atypical P-NENs	Hypoenhancing pattern		Hypovascular	Hypodense in arterial phase, possible enhancement in portal and late phases	Isointense or slightly hyperintense on T2-weighted sequences No strong restriction at DWI Hypointense in arterial phase, possible enhancement in portal and late phase
	Calcified pattern	Hyperechoic areas with prominent posterior acoustic shadowing		Hyperdense areas with high HU values	Signal void artefact
	Intraductal growth				Filling defect at MRCP Possible enhancement after contrast media
	Intravessel growth		Intravessel vascularized thrombus	Intravessel vascularized thrombus	Absence of flow void artefact Intravessel vascularized thrombus
	Cystic pattern	Anechoic lesion delimited by wall and possible inner septa	Hypervascular enhancement of wall and septa	Hypodense cystic portion Hypervascular enhancement of wall and septa	Hypointense in T1W, hyperintense in T2W Hypervascular enhancement of wall and septa

Small neoplasms are usually more homogeneous, before and after contrast media injection

Large neoplasms are usually more inhomogeneous, both before and after contrast media, due to the possible presence of necrotic and/or hemorrhagic areas

## Nuclear medicine techniques

The majority of P-NENs (about 70%), with the exception of insulinomas and poorly differentiated neoplasms, are usually characterized by lots of somatostatin receptors (SSRs) along the cell membrane, and this is the reason why functional imaging with somatostatin analogs is able to clearly show these neoplasms [40–42].

Somatostatin receptor scintigraphy (SRS), also called OctreoScan due to the first commercially available somatostatin analog called Octreotide, is based on the high affinity of synthetic somatostatin analogs for tissue expressing SSRs, such as most NENs.

This technique gives a scan of the whole body, allowing the detection of primitive tumor and metastatic lesions. Moreover, SRS gives functional information based on SSRs expression by the tumor, also contributing to the correct selection patients’ therapy with somatostatin analogs and/or radiometabolic therapy [2, 43]. Nevertheless, SRS has the intrinsic limit of basing itself only on the tumor SSRs expression. Non-specific uptake in inflammatory tissue or normal organs and poor intrinsic spatial resolution are two recognized limitations of SRS. Often, poorly differentiated NENs and insulinomas are not identified during SRS, and it does not give anatomical or surgical resectability information [2, 44].

Fused positron emission tomography (PET)/computed tomography (CT) is a recent imaging technique that provides functional information for PET study and anatomic details for CT study. PET/CT provides help in depicting pancreatic tumors and distant metastases, in performing pre-operative staging, and in monitoring treatment response [40].

A new PET tracer, 68Ga-DOTATATE, a new somatostatin analog combined with gallium (with other somatostatin analogs labeled with gallium), was recently introduced to nuclear medicine in the NENs study, and several investigations showed that it has greater sensitivity than OctreoScan in detecting P-NENs, in particular the small ones [45–47]. As a consequence, PET with gallium could be used in tumor detection and staging, and in selecting the correct treatment and therapy for patients.

Another radiopharmaceutical used in PET imaging is fluorodeoxyglucose (FDG), because, usually, tumoral cells have high glucose metabolism, and, as a consequence, they show FDG uptake. Generally, well-differentiated, slow-growing NENs demonstrate little or no FDG uptake, due to their low metabolic activity, whereas poorly differentiated NENs and metastases from P-NENs, which rarely express somatostatin receptors, are well depicted during FDG-PET. For the depiction of P-NENs, FDG PET has a sensitivity of 53–57% [40, 41, 48–50]. For these reasons, FDG PET/CT can play a role in discerning well- and poor-differentiated neoplasms because, with the increase in aggression and malignancy potential of the tumor, there is an increase in the glucose uptake by the neoplasm. FDG activity on PET is related to tumor progression and increased mortality [45, 51, 52].

Kayani et al. [42] reported that high-grade and poor-differentiated NENs show greater FDG uptake, whereas low-grade and well-differentiated tumors demonstrate greater 68Ga-DOTATATE uptake.

## Interventional radiology

Imaging methods, such as transabdominal ultrasound, endoscopic ultrasound, and CT, can also be used to guide fine needle aspiration or fine needle biopsy for cytological and/or histological confirmation of P-NENs.

Moreover, in NENs, interventional radiology could play an important role, especially in the control of non-surgical cases of disease.

Local treatment strategies such as embolization, chemoembolization, and targeted radionuclide therapy are increasingly being used in case of metastatic liver disease; moreover, local ablative techniques such as radiofrequency ablation (RFA) and cryotherapy are also important tools [53, 54].

**Fig. 9:**
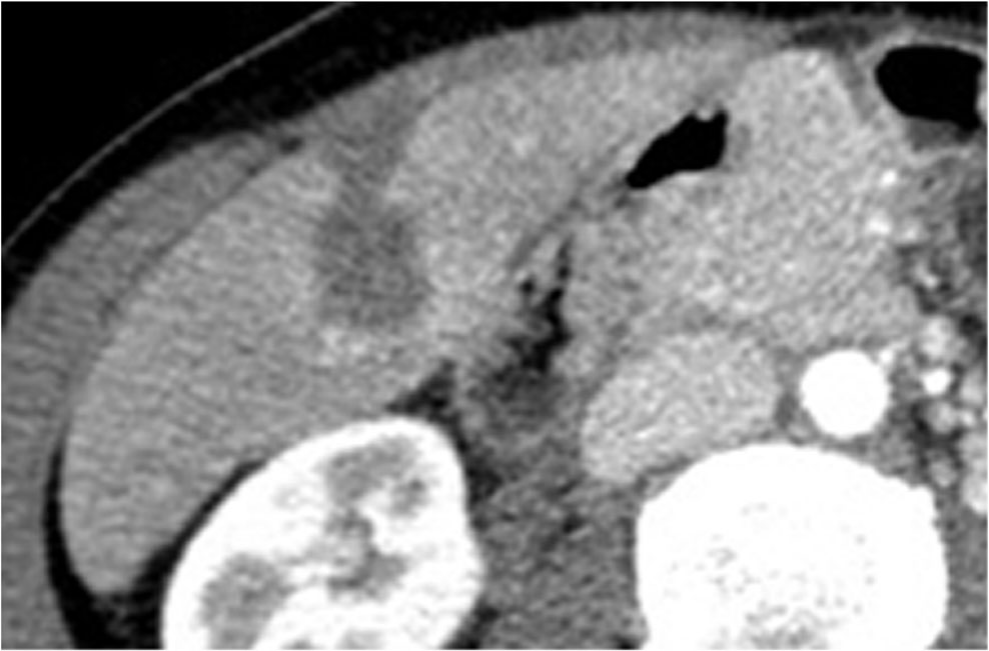
Thermolesion post-radiofrequency ablation (RFA). CT examination: hypodense avascular area in the right liver lobe (segment VI) related to coagulative necrosis post-RFA of small neuroendocrine metastasis

Usually, in case of P-NENs, the most relevant use of interventional radiology is for liver metastases treatment. Indications for imaging-guided RFA have been reported [53, 55]. During imaging after local ablative treatment, the ablation area (Fig. 9) has to be typically hypodense, with no enhancement during dynamic phases due to coagulative necrosis.

## P-NENs treatment

P-NENs treatment should be individualized for every patient and planned by a multidisciplinary group.

The first treatment for P-NENs is radical surgery, and in patients with advance disease and/or metastatic disease, a multidisciplinary approach should be linked to surgical methods [2].

Surgery is the treatment of choice for both primary tumors and metastatic lesions if eradicable, and this is why differential diagnosis with pancreatic ductal adenocarcinoma is very important, due to the completely different treatment choice and consequent prognosis.

In case of functioning neoplasms, surgery should be mandatory due to the symptoms complained by patients, also in case of small lesions, whereas in case of small (< 2 cm) non-functioning neoplasms well differentiated and with a low grade, nowadays it is possible to use a “wait and watch” follow-up, in order to avoid mortality and surgery complications in patients with lesions with a very low malignancy rate.

In some cases, for example in local advanced disease, it should be useful to follow a “neoadjuvant therapy” with somatostatin analogs and/or metabolic radiotherapy to downstage the disease and then apply surgery.

In case of metastatic disease, there are several possibilities, such as chemotherapy, therapy with somatostatin analogs, metabolic radiotherapy, and local treatment, the latter with interventional radiology, such as RFA and trans-arterial chemoembolization.

## Conclusions

Pancreatic neuroendocrine neoplasms (P-NENs) are typically hypervascular, but they could present with atypical features. At imaging, the atypical presentations of P-NENs are less frequent than typical presentations, but they could be very heterogeneous and hard to characterize, because of peculiar pathological changes, causing possible misdiagnosis. As a consequence, it is very important for radiologists to know all of the possible P-NENs presentations forms.

## Abbreviations

NEN: Neuroendocrine neoplasm
P-NEN: Pancreatic neuroendocrine neoplasm
RFA: Radiofrequency ablation
